# An Approach to Assessing Multicity Implementation of Healthful Food Access Policy, Systems, and Environmental Changes

**DOI:** 10.5888/pcd11.130233

**Published:** 2014-04-24

**Authors:** Laura Oliven Silberfarb, Sonja Savre, Gayle Geber

**Affiliations:** Author Affiliations: Laura Oliven Silberfarb, Gayle Geber, Hennepin County Human Services and Public Health Department, Minneapolis, Minnesota.

## Abstract

Local governments play an increasingly important role in improving residents’ access to healthful food and beverages to reduce obesity and chronic disease. Cities can use multiple strategies to improve community health through, for example, land use and zoning policies, city contracting and procurement practices, sponsorship of farmers markets and community gardens, and vending and concession practices in parks and recreation facilities. With 41 cities in the Hennepin County Human Services and Public Health Department jurisdiction, the county undertook to measure the extent to which cities were engaged in making policy, systems, and environmental (PSE) changes to increase residents’ access to healthful food. The results revealed that some cities, particularly those with higher resident demand for healthful food, are making nationally recommended PSE changes, such as sponsoring farmers markets and community gardens. Cities have moved more slowly to make changes in areas with perceived negative cost consequences or lesser public demand, such as parks and recreation vending and concessions. This article describes the assessment process, survey tools, findings, and implications for other health departments seeking to undertake a similar assessment.

## Introduction

The Centers for Disease Control and Prevention (CDC) and other major public health stakeholders have identified local governments as key players in the effort to create healthier communities, increase access to healthy foods and beverages, and reduce rates of obesity and chronic disease. CDC notes that many factors that “influence our health are created, managed, and maintained by local governments” (1). CDC and other federal agencies, national preventive health authorities, and leading municipal and planning associations have all issued formal recommendations, tool kits, or action guides that local governments and communities can use to implement policy, systems, and environmental (PSE) changes to increase access to healthful food. Despite the abundance of recommendations suggesting PSE change, it is not clear to what extent cities are interested in or are making changes at the local level, particularly in smaller suburban areas and rural towns.

## Methodology

The Hennepin County public health jurisdiction includes 41 cities and excludes 4 cities in the county — Minneapolis, Bloomington, Edina, and Richfield — that have their own boards of health. Our healthy food policy assessment focused on the largest 20 cities in the Hennepin County public health jurisdiction, excluding the 4 cities noted, ranging in population from 2,400 to 76,000 people. The combined population for the 20 cities totaled 569,254.

The first step in the assessment process identified the universe of recommended local PSE strategies that cities could use to affect residents’ access to healthful food. Our goal was to develop a uniform set of local PSE changes for healthful food access to use as the basis for the assessment in interactions with officials in all cities. CDC and other federal agencies, national prevention authorities, and leading municipal and planning associations have all issued formal recommendations, tool kits, or action guides providing information local governments can use to implement PSE changes to increase access to healthful foods and beverages. Yet no one set of recommendations offered the detail we needed to understand how we could best provide technical assistance to local government agencies when the assessment was completed. We chose *Recommended Strategies and Measurements to Prevent Obesity in the United States* (1) as the basis for selecting the PSE changes in the assessment; we further developed the list by adding more specific strategies from the national tool kits and action guides (2–7). Although the specifics of the PSE recommendations in each of the guides varied, most of them fell under general categories including:

- Increasing the availability of healthful foods and beverages in vending and concessions
- Establishing procurement and contracting policies for healthful food
- Increasing the production and availability of food from farms
- Increasing the number of community gardens
- Decreasing opportunities for the purchase and consumption of sugar-sweetened beverages
- Establishing workplace breastfeeding policies
- Increasing the availability of supermarkets and healthful- food retailers in underserved neighborhoods
- Increasing access to cool drinking water in public places
- Increasing the availability of healthful food and beverage options in restaurants
- Establishing food councils

From our review of national action guides, we created a range of PSE options and practice changes for local agencies to use as the framework for our assessment. We used a 2-stage approach to collect information from the cities in 2012. First, we sent an electronic survey to a point person in each city and then conducted 36 in-person and telephone interviews.

Public health department staff supplied the interviewer with names of municipal staff we previously worked with in the target cities. These point people identified others to be surveyed or interviewed. One interviewer conducted all interviews. For 15 cities, the interviews were conducted in person. Interviews with the remaining 5 cities were conducted by telephone at the convenience of the point people. Several of the point people brought their wellness staff to the interview. In one city, 5 people participated in the interview.

All data presented in the analysis were collected from the interviews. Data were categorized according to an a priori framework based on policy recommendations from national reports. Results were tabulated on the number of cities that had implemented any of these strategies. Open-ended questions were content-analyzed to identify common themes.

### The survey process

We created an electronic survey with a checklist of possible PSE options, asking officials to identify changes they had made in their city. We added a Likert scale of interest in improving healthful food access to get an approximate sense of city officials’ readiness for change. We grouped the checklist of PSE options by city departments likely to complete certain sections of the survey. Knowing that the survey would require input from multiple city departments, we identified a point person in each city to help streamline the collection of information. To help them efficiently collect information from the other departments, we programmed the survey instrument to allow multiple users to input into the same record. We sent a separate letter in advance of the survey to explain the effort and to promote survey completion and sent 2 additional reminders over a 2-week period. Of the 20 cities, 10 responded. Some of the surveys contained relatively little information. It appeared that some of our city contacts may not have shared the survey with other departments to collect the full breadth of requested information on city activities. Because of the low response rate from the surveys, these data were not analyzed.

### The interview process

The second stage of the assessment was designed to collect more in-depth information through in-person and telephone interviews with city officials. One of the first steps was to decide which official to interview in each city. Cities vary in their organizational structure. For crosscutting city initiatives, we selected economic development and planning department officials as those most likely to be knowledgeable. We also made the decision to conduct separate interviews with parks and recreation directors because these operations are typically run independently and are often located in recreation centers away from City Hall. In addition, parks and recreation interviews involved much more specific and detailed information on vending and concession operations, contract practices, and food policies. Eleven of the 20 cities in the assessment had a separate parks and recreation department. In the 5 smallest cities with populations of approximately 5,000 and few staff members or programs, we conducted telephone interviews with 1 official. We achieved 100% participation in the interview process with at least 1 official in all 20 cities.

We designed the interview protocol as a tool to collect data on both the PSE changes and factors that affected the city official’s decision-making process. Originally we designed the interview protocol to build on the information we gathered in the electronic survey of PSE options. With only 10 cities responding to the electronic survey, and because of our concern that some of these surveys were incomplete, we began each interview by quickly reviewing the checklist of possible strategies with the city officials. Once we identified the PSE changes made in each city, we used the interview to delve deeper into issues they faced and to identify barriers to taking action.

By using our checklist of PSE options, we structured the interviews along broad topic areas including community gardens, farmers markets, healthy city halls (procurement, contracting, and meeting policies), and parks and recreation food and beverage concessions and vending. We also designed an open-ended section where cities could add other PSE changes they had instituted such as new water spigots in City Hall, passage of a healthy community resolution, and backyard livestock zoning changes. We did not include questions in our interviews about nationally recommended PSE changes related to healthy corner stores and supermarket availability because Hennepin County had recently collected this information through a different survey effort. Nor did we cover breastfeeding policy because state law already covers breastfeeding in work-related settings.

## Results

Cities were selective in their implementation of the national PSE recommendations. Although 16 of the 20 cities we assessed were at least somewhat interested in improving access to healthful foods, they varied widely in their use of local authority to make changes. Among the PSE strategies (Table), cities were most likely to sponsor or make land available for farmers markets (11 cities) and community gardens (12 cities). Two cities made healthy City Hall organizational changes using their contracting or purchasing authority to improve the food environment. No city made any changes related to restaurant foods and beverages, and none had instituted a food council. We found the greatest variation among the parks and recreation system approaches to improve the nutritional content of food and beverages in vending and concessions: 5 cities had worked intentionally to expand healthful food and beverage options in their community centers, while others made no changes. Three of these 5 also made changes to offer healthier food and beverage vending. No city parks and recreation system implemented a uniform nutrition standard or other policy that applied to all foods and beverages in vending and concessions.

**Table:** Number of Cities Implementing Healthful Foods and Beverage Policy, System, or Environmental (PSE) Strategies, Hennepin County, Minnesota, 2012^a^

Strategy Type	Cities With PSE Strategy
**Citywide**	
Community gardens	12 of 20
Farmers markets	11 of 20
Healthy city halls	2 of 20
Policy regulating healthy food and beverages in restaurants	0 of 20
Food councils	0 of 20
**Parks and recreation strategy^b^**	
Concessions	5 of 16
Vending	3 of 12
Uniform policies	0 of 10

a Study included 20 largest cities in county, excluding Minneapolis, Bloomington, Edina, and Richfield.

b Because not all cities have parks and recreation concessions or vending, the denominator changes for each row.

Overall, it seemed that the cities that created greater access to healthful foods and beverages — particularly with community gardens and farmers markets — were those that experienced the greatest demand from residents. It also appeared that the presence of wellness committees in city halls might have supported progress toward greater access to healthful foods and beverages.

### Community gardens and farmers markets

Cities predominantly chose sponsorship of community gardens (12 cities) and farmers markets (11 cities) to improve healthful food access for residents. City officials noted that public demand for community gardens (9 cities) and farmers markets (6 cities) was a key motivating factor. At least 2 officials noted that their elected officials were especially enthusiastic about these approaches because gardens and markets were popular community gathering places. Three officials also observed that recent immigrant populations, particularly Southeast Asians and East Africans, were interested in owning community garden plots. In 4 instances, cities also chose to sponsor community gardens to better serve residents in multiunit rental housing who would not otherwise have the opportunity to grow their own food. At least 3 cities noted that the cost of bringing in and maintaining a water supply for the gardens was a challenge. None of the cities were equipped to accept Electronic Benefit Transfer cards from the Supplemental Nutrition Assistance Program, but 2 expressed an interest in doing so.

### Healthy city halls

Two cities had changed their vending machine contracts to secure a “healthy food and beverage” machine in their city hall; however, none of the cities had implemented purchasing or contracting policies for foods and beverages. Ten cities had employee wellness committees that had been actively involved in health-related activities. Seven of these were informally moving toward offering more healthful foods and beverages at meetings. However, no city had implemented a policy of offering healthful foods at meetings. In general, officials seemed reluctant to set a formal food policy and were more inclined to influence the environment by promoting healthful options rather than regulating change.

### Parks and recreation concessions and vending

Hennepin County cities have invested heavily in building a comprehensive parks and recreation infrastructure with an impressive array of community centers, indoor ice arenas, aquatic centers, baseball fields, and other athletic facilities. Parks and recreation systems often operate concession stands or vending machines in these complexes. To get a sense of the scope and potential impact, we asked parks and recreation directors to calculate the number of patrons they serve through their city athletic facilities with concession stands. Sixteen city parks and recreation systems that operate or lease concession stands or cafes had a combined 4 million patron visits annually.

Twelve city parks and recreation systems sold foods and beverages through vending machines. Only 1 city had implemented a formal vending nutrition standard for foods and beverages based on the National Automatic Merchandising Association “Fit Pick” nutrition standard (8). This city required that 25% of the foods and beverages sold in vending machines meet the Fit Pick standard, which requires that foods and beverages have less than 35% of total calories from fat, less than 10% of calories from saturated fat, and less than 35% of total product weight from sugar. One other city set a 25% goal of healthful vending machine foods and beverages, but did not choose to define “healthy” or set a nutrition guideline standard. One other city that owns and stocks its own vending machines added some healthier items. In addition, 5 cities had intentionally expanded the options sold in at least some of the concession stands to create a more healthful menu. Cities added items like fresh sandwiches, carrots, and yogurt. No city had implemented a parks and recreation-wide nutrition standard that applied to 100% of the foods and beverages sold in vending machines and concessions.

Our interviews with parks and recreation officials gave us insight into the factors that affected their decision making. Although parks and recreation officials are largely supportive of the idea of improving access to healthful food, they also identified numerous barriers to implementing formal nutrition standards that apply to all foods and beverages.

Principal barriers identified by parks and recreation and other officials included:

- Fear of losing profits and revenue. Parks and recreation facilities are typically run as enterprise funds where concessions and vending fund core operational costs. Enterprise-funded centers must generate profits or make cuts in their operations and programming. At least 3 cities noted that the wholesale costs for certain healthful options were sometimes more expensive or that fresh foods have a higher likelihood of perishability, which is a financial liability. One city closed its park concessions enterprise operation because of a lack of profitability.
- Lack of control over concessions. Of the 16 cities with concessions, 7 have yielded control of at least 1 of the concession stands to a sports association. For many hockey and baseball teams, this is an important source of revenue.
- Lack of control over vending. In 11 of the 12 cities with parks vending, the vending company controlled the selection, display, and price of food and beverage options. In the 1 exception, the city buys its own products independently and does not contract with an outside vendor. Vending contracts are typically locked in for multiple years, especially beverage contracts with major national beverage companies.
- Inconsistency of demand. Public demand for more healthful foods and beverages is increasing, albeit gradually. Although the public is asking for healthier options, at least 6 parks and recreation directors pointed out that the public still expects less healthful options on the menu. One official noted that when families come to the swim park for a special occasion, they want to treat their kids with an ice cream or candy.
- Reluctance toward formal policy implementation. City officials are reticent to implement formal policies that restrict options and prefer the more flexible approach of expanding healthful food and beverage options. In 5 interviews, a city official volunteered that while the city is moving toward expanding healthier food and beverage options in City Hall or in the parks and recreation system, they did not want to formalize the approach through a written policy.

## Lessons Learned

Public health departments interested in conducting a similar healthful food and beverage PSE assessment may want to consider the following:

- If possible, embed the survey tool of PSE options in the interview process instead of conducting a written survey as a separate step. By conducting interviews, we achieved a 100% response rate from the 20 cities and obtained significantly more in-depth information than a survey alone would have yielded.
- Try to identify the most knowledgeable city official for crosscutting city initiatives. We most often found city planning and economic development officials to be helpful.
- Conduct interviews with parks and recreation officials separately from interviews with other city officials. Parks and recreation interviews were more technical; they included detailed information on food and beverage purchasing practices and enterprise operations related to vending and concession practices. This information was valuable given the significant number of patrons using athletic facilities and the important potential role they play in improving access to healthful food for residents, especially youth.
- Structure the interview protocol to include information on changes in practices, especially if you anticipate that few cities in your jurisdiction have implemented policy change.
- Share your assessment report with cities after the assessment has been completed. City officials are sometimes reluctant to be change leaders. Many cities asked us for the report to learn more about neighboring city initiatives and progress.

The Hennepin County Human Services and Public Health Department is using the assessment results to identify cities interested in improving access to healthful food and as baseline data to measure future change. The results will also help the county address the barriers revealed through the assessment and develop the technical assistance necessary to institute improvements countywide.

This research has several limitations. We did not establish psychometric properties of our data collection instruments. Our study design does not allow us to determine with certainty which factors most influenced greater access to healthful foods and beverages. Finally, there is limited generalizability of the findings to other locations.

## Conclusion

Conducting a municipal PSE assessment is a valuable first step for local public health departments seeking to improve residents’ access to healthful foods and beverages. The assessment can help public health departments understand not only where cities are on the continuum of possible PSE change but also identify barriers to change. The assessment will also enable public health departments to identify crosscutting themes and tailor technical assistance to meet the needs of cities interested in further improving resident access to healthful food.
